# The impact of need on distributive decisions: Experimental evidence on anchor effects of exogenous thresholds in the laboratory

**DOI:** 10.1371/journal.pone.0228753

**Published:** 2020-04-01

**Authors:** Bernhard Kittel, Sabine Neuhofer, Manuel Schwaninger

**Affiliations:** Department of Economic Sociology, University of Vienna, Wien, Austria; Universität Heidelberg, GERMANY

## Abstract

Giving more to those who need more has an intuitive appeal for determining the just allocation of resources. The need principle is considered one of the three major principles of distributive justice. In contrast to equality or equity, however, evidence on the adherence to the needs principle rests mainly on stated instead of revealed preferences. In this paper we present an experimental design that exogenously assigns objective, heterogeneous need thresholds to individuals in small laboratory societies structured by a three-line network. The data reveal that a large proportion of individuals respond to others’ need thresholds, but at a declining rate as thresholds increase. The equal distribution marks a discrete drop in the need satisfaction rate: Need thresholds above the equal distribution are less frequently satisfied. We conclude that others’ needs are weighed against self-interest and equality. Our results provide evidence that distributions may be socially justified on grounds of the need principle.

## Introduction

### Needs as thresholds

A shared understanding of distributive justice and fairness among a community’s members is fundamental for social order [1]. The principle of supporting others in need fosters the development of solidary communities and has contributed to the establishment of the modern welfare state [2, 3]. The satisfaction of needs is one of the major normative principles of distributive justice, next to equity and equality [4–6], and has been the focus of substantial philosophical reflection [7–10].

According to the need principle an allocation is just if the individual payoffs satisfy the needs of all members ([11], p. 149). However, assessing whether individuals consider others’ needs is surprisingly challenging due to the impossibility to objectively identify relevant needs [9]. In the philosophical discussion, a common understanding is that needs cannot be defined in terms of the possession of, or access to, a set of commodities, but have to be considered as “requirements for ‘the opportunity for a full life’” ([12], p. 342). This conception implies that needs extend beyond physical functioning and include social participation in society [7].

To further specify the concept of needs, the “capabilities approach” claims that there exist some thresholds for the provision of means below which the states and activities constitutive for a dignified life cannot be realized [13]. In this vein, the United Nation’s Human Development Index captures various capabilities. Nevertheless, any attempt to provide a complete and coherent list of capabilities is bound to fail due to the impossibility of identifying universally relevant capabilities, the numerous interdependencies between single needs, and the problem of adjudicating between different needs and the needs of different persons [14]. These ambiguities fundamentally hamper not only the development of an empirically informed theory of need, but also make the relevance of the need principle difficult to test in real-world contexts.

We take a more direct approach and test the recognition of the need principle in social interaction in a monetarily incentivized laboratory experiment. We implement individual need thresholds, which must be satisfied for survival in the game, in a three-person, strong-power network [15–17] and study distributive outcomes in social interactions. We focus on triads as this is the minimum group size necessary for the emergence of social properties, such as norms, and for the generation of shared values underlying norms, such as principles of justice [18]. We additionally induce a strong power differential by arranging the three group members in a hierarchical “three-line” network structure of the form *A*–*C*–*B*, where *C*, the central position, has more structural power because *C* can choose with whom to negotiate and agree. This enables us to relate our results to a clear reference prediction [19]. If needs serve as a criterion for the distribution of resources, need thresholds should form particular focal points for individual allocations.

### Related literature

Our experimental results contribute to various scholarly literatures. Needs are relevant in hypothetical decision tasks [20, 21] and need satisfaction ranks high among justice attitudes in population surveys [22, 23]. In incentivized laboratory experiments, a combination of a floor constraint and the maximum average is the most popular choice [24–29]. This approach reflects the idea of inclusion but does not take into account the heterogeneity of needs. In monetarily incentivized dictator games, dictators give more to recipients who are poor or live in a poor country [30, 31]. These studies exploit subjects’ knowledge about deprivation to induce them to raise transfers, which is basically a humanitarian act. However, being poorer than others does not necessarily reveal information about individual needs and neither does it include any reference to a shared norm of need-based justice. These studies strongly suggest that people hold social value orientations. A test involving real needs finds that people differentiate between basic and instrumental needs [32].

In contrast to the justice-related literature focusing on the relevance of internalized norms in distributional decisions, most traditional approaches in Social Exchange Theory assume that actors are purely self-interested and they predict that actors choose the exchange relation that maximizes their own payoffs. This framework explains relative allocations by the structure of the network, which attributes negotiation power to nodes by the number of connections to other nodes [33, 34]. For the three-line network of the form A–C–B, on which we focus, all models assuming narrow self-interest predict that the powerful, central, node C obtains most of the resource (between 1 and 5/6), the agreeing partner obtains the remaining share (between 0 and 1/6), and the third player is left with zero [35]. Newer approaches integrate the possibility that actors hold fairness preferences toward all members of the exchange network, and not just immediate interaction partners [36]. The evidence currently available on the effect of social value orientations on distributional outcomes in social exchanges is mixed, however [37, 38].

### Aims: The effect of need thresholds as a structural condition

We presume that in allocative decisions subjects’ material interests and normative persuasions provide potentially conflicting behavioral incentives [39]. Induced value theory assumes that by financially incentivizing a decision problem, the goal of maximizing one’s own income overrides other motives [40, 41]. Many scholars assume the gain goal–the maximization of one’s own payoff–to be the “natural state” of individuals [42] since subjects are assumed to voluntarily participate in laboratory experiments in order to earn money. There is ample evidence, however, that subjects also hold other-regarding preferences [43–46] and that these are closely related to justice attitudes [47]. Seen through the lens of the model of frame selection [48], self-interest is not always sufficiently strong to override internalized justice norms, such as the moral value of caring for others, that is, satisfying others’ needs. For example, it has been shown in the three-line network that dyads who are given the opportunity to allocate part of their resource to a third player actually do so despite contrasting incentives [38].

In this paper, we do not aim to study social, i.e. other-regarding, preferences per se. By definition, distributive justice involves comparison with others and the criteria by which it is judged is in the eye of the beholder [49, 50]. By merely observing incentivized behavior in the laboratory it is difficult to disentangle different sources of motivation, such as social preferences. Instead, we focus on the behavioral responses to *structural* variation in need thresholds: To what extent and under which conditions are others’ needs recognized as a reference point for distributive decisions in the group?

In order to assess the influence of individual need thresholds on behavior expressed as exchange patterns, we study their effect in an experimental design that contrast the recognition of needs with self-interest and the equality principle. First, the intensity of conflict between self-interest and others’ need satisfaction increases with others’ need thresholds. Hence, we are generally less likely to observe the satisfaction of others’ needs, the higher their need thresholds are. Assuming self-regarding preferences, own needs are always put upfront and subjects will not compromise on them. Second, the need principle may enter into rivalry with other justice principles, most notably equality, which is the modal outcome in distributive decisions without further information [4]. In this study, equality relates to the distribution of a joint resource, without consideration of any further aspects related to the decision context. Need, in turn, is interpreted as the resources required to reach a position in which subjects can further accumulate resources. Satisfying others’ needs thus means to place them in a position to continue earning money, which is a value in itself on top of the instrumental value of earning money [51, 52]. If need thresholds exceed the share allocated by an equal distribution, the need principle and the equality principle conflict. Therefore, if individuals adhere to the stated conception of equality, we expect a sharp drop in need satisfaction when the need threshold is raised above the equal share of the resource (see *Methods and Materials* for the formal model).

### Measures: Need thresholds

We measure behavioral responses to individual need thresholds in a two-stage laboratory experiment by means of observing the outcome of *dyadic negotiations* concerning the allocation of a limited resource in a three-person network, a setup which builds on a long experimental tradition in social exchange theory [33, 34].

In order to measure the concept of *need thresholds* in a laboratory experimental setting, we simplify the multi-dimensional concept of capabilities to a one-dimensional, quantifiable property. The closest representation of the concept of “survival in dignity” [13] in a laboratory setting is the capability to proceed to a further stage in the experiment, similar to a board game in which participants drop out if they fail to meet a necessary requirement for continuation. While the intrinsic value of the opportunity to earn additional payoffs is clear, the specific extrinsic value of the second stage is relatively uncertain. Furthermore, we limit competition between subjects’ need claims by setting the sum of thresholds lower than the total endowment to be distributed in all but one constellations (see Table 1). There is no financial incentive to satisfy others’ needs.

**Table 1:** Treatments: Thresholds and descriptive measures.

Scenario	Thresholds	Sum	NSR-N	Mean Range of profits
A1	c5–0–9	14	0.63	7.36
A2	c1–9–5	15	0.41	7.7
A3	c5–1–12	18	0.3	9.61
A4	c9–5–1	15	0.52	8.61
A5	c0–0–0	0	1	7.47
A6	c5–9–1	15	0.53	8.59
A7	c5–5–5	15	0.52	6.63
B1	c5–1–1	7	0.78	4.5
B2	c5–9–5	19	0.59	5.31
B3	c5–5–12	22	0.41	8.5
B4	c5–5–1	11	0.69	6
B5	c5–12–12	29	0	10.75
B6	c5–9–9	23	0.28	8.44
B7	c5–5–5	15	0.44	6.72

The table shows the distributions of thresholds–referred to as “scenarios”–participants were confronted with in a lab session. The resource per period and network was 24 points. Treatment A (heterogeneous thresholds): N = 192 observations on subject level per scenario (i.e. N = 64 on network level). In column “thresholds” the letter “c” denotes the central position. Treatments–combinations of scenarios–varied between sessions. As a robustness check the sequence of scenarios was altered to A7-A4-A6-A3-A2-A1-A5. We control for time effects in Table A in S2 File. Overall, the results are robust, with the exception of scenarios c5-5-1 and c5-0-9, which cease to be statistically significant in comparison to c5-1-1. This is not surprising, as in all three scenarios thresholds are relatively easy to satisfy. Column “NSR-N” refers to the frequency of need satisfaction on the network level. Treatment B (constant thresholds of central player): N = 96 observations on subject level per scenario (i.e. N = 32 on network level). Scenario “B5” will be excluded from analysis unless specifically stated, as the resource is smaller than the sum of thresholds. “Mean range of profits” is the absolute difference between all three players’ incomes. It is a measure of profit inequality within the network–the lower it is, the closer is the distribution to the equal three-way split.

A distribution of allocations is considered to be just according to the need principle if the allocation to each subject is equal to or larger than her individual threshold in *stage one* (St1) and, thus, all members of the network are admitted to participate in *stage two* (St2) and earn additional income. The *need satisfaction rate* (NSR) expresses the frequency of need satisfaction on the network level (NSR-N) and on the individual level (NSR-I).

### Experimental design

In St1, the group-level allocation stage, subjects were assigned to nodes in a three-line network of the form *A*−*C*−*B*, and negotiated the allocation of a resource of 24 points to the three players by sending each other numeric proposals of distributions in the format {*C*,*A*,*B*}. Any number of proposals was allowed within three minutes along connecting edges of the network. No text messages were possible and only one binding agreement could be made between two of the three subjects. An agreement was concluded when the recipient of a proposal clicked on the “accept” button (see “Details on procedures” below and *“Experimental Instructions”* in S4 File for details and screenshots). If a subject earned at least her designated threshold (see Table 1 for assigned thresholds), she was admitted to St2, the real-effort stage, where she could individually and independently earn additional points in a set of tasks. If she did not reach the threshold, she kept all points obtained in St1 but could not earn anything in addition in St2. Points earned in St2 were added to those of St1 for the total individual payoff. Individual performance and earnings in this stage were private information and did not affect the others’ payoffs. The tasks in St2 varied, and included summing or subtracting numbers, counting letters in sentences, and answering randomly sampled general knowledge questions. Subjects were informed about the diversity of the tasks beforehand.

#### Treatments

We varied the combinations of thresholds *t* –referred to as *scenarios*–systematically within sessions. Treatment A: Individual thresholds were heterogeneous and varied for all individuals. Treatment B: Thresholds were heterogeneous as well, but the threshold of the central position was uniformly set at 5 points. Columns 1 to 3 in Table 1 report the selection, sum, and sequence of thresholds in both treatments. In each session, subjects participated in seven scenarios, i.e. seven sequences of St1 and St2. Network positions were fixed. After each scenario, subjects were randomly matched as strangers (hence never interacting more than once) into new three-node networks. Thresholds were always common knowledge. A practice period using thresholds of 5, 1 and 9 was played without payoffs in order to familiarize participants with the procedure and offer the opportunity to ask questions regarding the functionality of the program interface and game.

#### Network structure

The three-line network produces variation in structural power in a very simple setting. Players negotiate the distribution of allocations in dyads, implying that the central player (C) is pivotal because she has two potential partners (A, B), whereas the other two players can only negotiate with the central player. One player is excluded from the decision by design, but may be allocated a share of the resource. Using this difference in power and limiting admissible exchanges (i.e., distributive decisions in the present context) to one per interaction sequence creates a competitive environment and, thus, generates a strong test of the relevance of need satisfaction in negotiations.

In addition we implemented the Social Value Orientation Slider Task [53], which translates distributive preferences, such as spite, self-interest, inequality aversion, efficiency concerns, or altruism, into a continuous scale and allows us to control for other-regarding preferences on a single dimension.

### Hypotheses

How does the introduction of need thresholds affect distributive decisions? The higher the Social Value Orientation scale (SVO), that is, the more pro-social players are, the more likely players will take others’ need thresholds into account. Thus, overall satisfaction of need thresholds should increase with an increase in aggregate social value orientations in the network.

H1: NSR-N is positively related to the aggregate SVO score of all players in the network.

However, with increasing need thresholds of other players, the share of one’s own income one has to sacrifice to satisfy others’ needs increases. For each level of the SVO score there is a point at which self-interest wins against the norm of need satisfaction. Hence, the willingness to satisfy each other’s need thresholds is expected to decrease with rising incompatibility of self-interests and others’ allocative claims.

H2: The higher player *i*’s need threshold, the lower the probability that this threshold will be satisfied (NSR-I is negatively related to the level of player *i*’s need threshold).

We expect that this effect will be much smaller–if present at all–for players in the agreeing dyad. One possible situation in which the peripheral player in the dyad might accept a share not satisfying her threshold may be the fear that the central player agrees with the other peripheral player on an even lower share for her. Once a dyad agrees on a distribution, the third player depends on the goodwill of the other two players. We expect that the higher the third players’ need threshold is, the less likely her needs will be satisfied. The third hypothesis thus refers to the tension between the need principle and self-interest.

H3: Third players’ NSR-I decreases at a faster rate than the NSR-I of the peripheral player in the dyad, for any increase in the need threshold.

If a need threshold exceeds the share corresponding to an equal distribution of the resource, the need principle is not only at odds with self-interest but also with the equality principle. The argument that relative disadvantage weighs more than relative advantage has been forcefully put forward [54]. Hence, we expect the willingness to satisfy need thresholds that exceed the allocation to self to drop sharply as the threshold surpasses the equal distribution of the available resource. Beyond the equal distribution a different mechanism is activated. Whereas the satisfaction of need thresholds below this point can be simply motivated by a concern for equal opportunity, the satisfaction of thresholds above this point additionally requires overcoming a distaste of disadvantageous inequality [55].

H4: The NSR-I is higher for thresholds below the equal three-way split than for thresholds above this level.

## Results: Needs matter

We find that a large number of individuals respond to others’ need thresholds. In fact, the distribution of needs affects negotiations systematically and the distribution of payoffs shifts towards the need thresholds. However, need satisfaction declines with increasing thresholds. The findings are in line with the interpretation of the equal distribution as a trigger point: We observe a substantial discrete drop in the need satisfaction rate for thresholds above the equal distribution in comparison to thresholds below that point.

**Fig 1:**
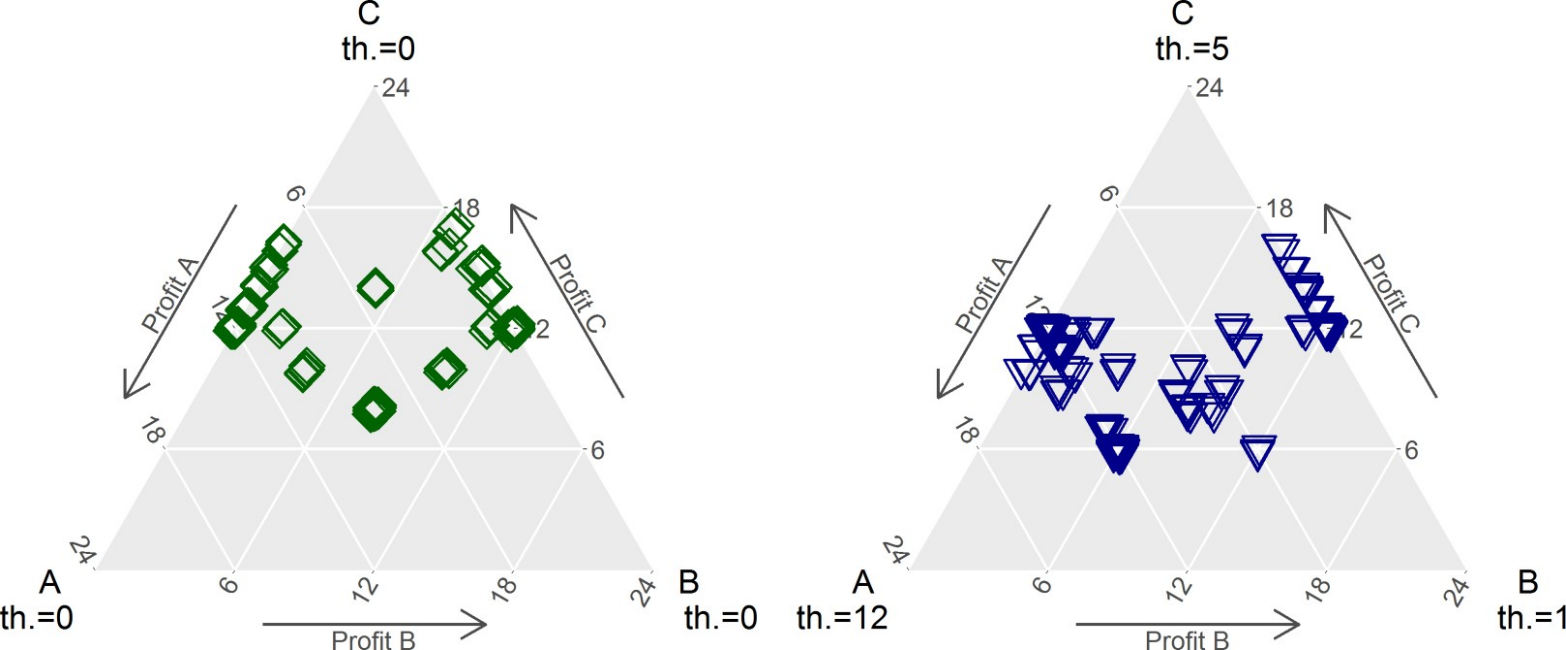
(A and B). Distribution of allocation agreements (accepted offers). Panel A: Scenario c0–0–0. Panel B: Scenario c5-12-1. The nodes of the graph denote the positions of the players: C is the central player, A and B are the peripherals. Thresholds are denoted below the label of the positions, whereby Panel A displays the scenario c0-0-0 and Panel B c5-12-1. The arrows aside the graphs indicate each player’s payoff: The closer a mark to the position, the higher her share of the allocation decision. There is a notable difference in clusters of allocations between the scenarios presented. The cluster at the centroid denotes an equal split between all three subjects. Marks are slightly jiggled in order to visualize local clusters of player positions.

Fig 1A and 1B show the two most extreme scenarios, which give an impression of the shift generated in the need satisfaction rate by the manipulation of need thresholds. We study various scenarios, denoted by *ct*_*C*_−*t*_*A*_−*t*_*B*_, whereby *c* identifies the central player. Panel A depicts the allocation of points in scenario *c*0−0−0 and Panel B shows *c*5−1−12, in which one peripheral player is attributed a comparatively high threshold. We see that in Panel A allocations are drawn both more to the top, i.e. to the central player, and to the focal points of equal three-way split and equal two-way split. In contrast, Panel B shows a larger cluster between the central and the peripheral player with threshold 12, and the accepted offers between the central and the peripheral with threshold 1 are drawn more upwards to the central position, indicating that thresholds are indeed used as focal points. Furthermore, there is a cluster of points just to the right of the focal point on the left axis of A’s payoff. The small distance to the axis indicates that the other peripheral’s threshold of 1 point was satisfied. The impact of need thresholds on behavior becomes even more apparent when contrasting payoffs. For peripherals, the mean payoff is 5.95 for a threshold of 1, and 7.66 for a threshold of 12, showing a qualitative difference in allocated shares between peripherals, taking into account the respective thresholds (see *“Exemplary distribution of profits*” in S2 File for more details on distribution of profits, individual income of positions and test of difference for Fig 1A and 1B).

### Test of hypotheses

We find evidence for an effect of social value orientations on distributions (*H1*). Table 2 shows that, controlling for the sum of need thresholds in a network, the aggregated SVO in a network has a strong positive effect on NSR-N. The higher the SVO, i.e., the more prosocial the members of a network are, the higher NSR-N. Compared to scenario *c*5−1−1, which is characterized by both the lowest symmetrical thresholds for both peripherals and the smallest sum of thresholds, all other scenarios have a lower NSR-N, whereby the higher and the more unequal the thresholds are, the larger are the negative coefficients.

**Table 2:** Logistic regression of aggregated SVO and scenarios on NSR-N on the network level.

	Coefficient	Stand Err	Lower 0.95	Upper 0.95	Odds Ratio
Sum of SVO in network	0,5142	0,1807	0,16	0,8684	1.6723
Scenarios (ref. c5-1-1)					
c5-5-5	-1,5175	0,3772	-2,2568	-0,7782	0.2193
c5-0-9	-1,039	0,4669	-1,9541	-0,1239	0.3538
c1-9-5	-1,9563	0,4549	-2,848	-1,0646	0.1414
c5-1-12	-2,4491	0,4895	-3,4086	-1,4897	0.0864
c9-5-1	-1,5032	0,4164	-2,3193	-0,6871	0.2224
c5-9-1	-1,4378	0,4541	-2,3277	-0,5478	0.2374
c5-5-1	-0,4872	0,1828	-0,8454	-0,129	0.6143
c5-9-5	-0,9046	0,2509	-1,3963	-0,4128	0.4047
c5-5-12	-1,6795	0,3374	-2,3408	-1,0183	0.1865
c5-9-9	-2,2494	0,2238	-2,6879	-1,8108	0.1055

Dependent Variable: NSR-N, 1 = all three thresholds satisfied; 0 = at least one threshold not satisfied. N = 576; scenario c5-12-12 is excluded, since NSR-N always < 1; Scenario c0-0-0 is excluded, since NSR-N always = 1. Standard Errors are clustered on the group level of the session, whereby one session consisted of either one or two independent groups of 12 individuals, depending on whether one or both treatments were implemented at the same time. See Figure A in S2 File for a plot of the predicted probabilities of the scenarios.

This result also holds for the share allocated to the third player, which in many cases covers this player’s need threshold. The Social Value Orientation (SVO) of the agreeing dyad, and foremost of the central player, exerts a positive effect on the third player’s NSR-I.

**Fig 2:**
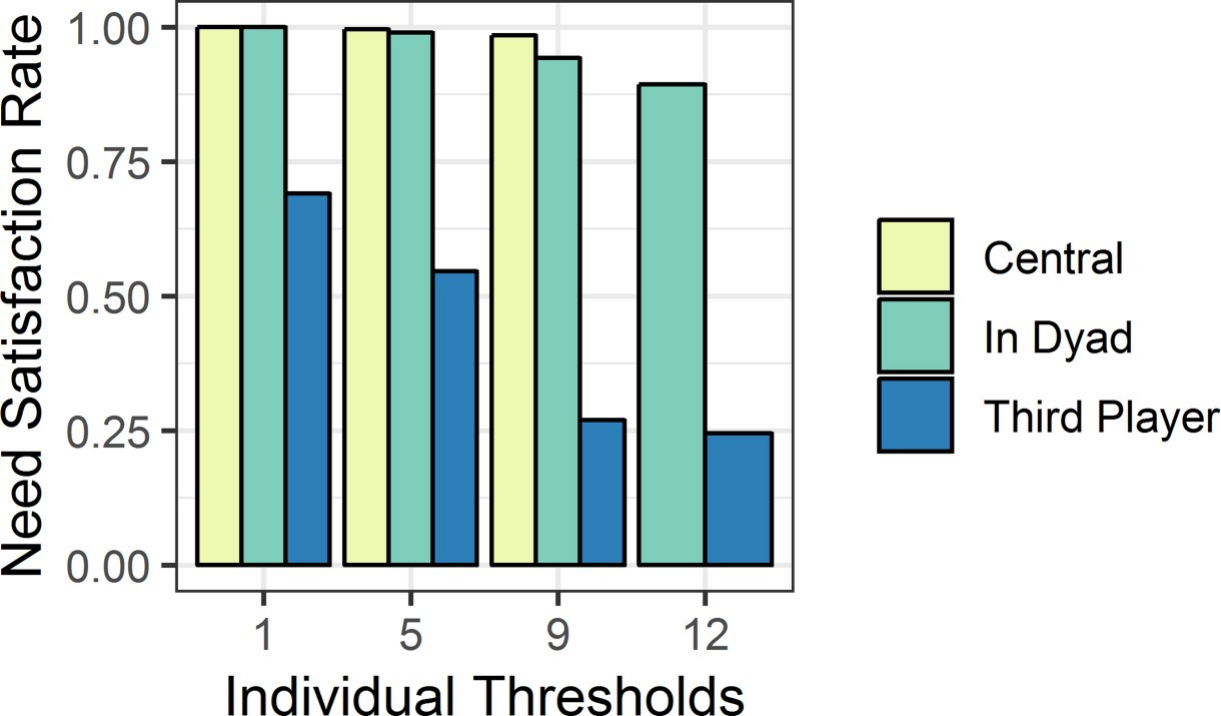
NSR-I by position over individual thresholds. The x-axis shows individual need thresholds, the y-axis the NSR-I for individual observations. The central player never was assigned a threshold of 12 points (see *Table 1* for list of thresholds). All scenarios with sums of thresholds smaller than the resource of 24 points are included. Columns are split by network position (central or peripheral) and by the “role” (in dyad or third player) a participant assumed in each period. The central player could not be excluded from agreements. A peripheral player either became the agreement partner, by being part of the dyad, or she became the third player, by being excluded from the agreement. Exclusion from the agreement does not imply that this peripheral player did not receive any payoffs from this period, because the agreeing dyad may allocate some share to her.

Next to the effect of social value orientations, we observe that NSR-I decreases with increasing individual need thresholds for both peripheral players (supporting *H2*), but in particular for the third player (supporting *H3*), as displayed in Fig 2. Central players’ needs are always satisfied if an agreement is reached. In two cases no agreement was concluded within the time limit of three minutes, resulting in the outcome that the central player’s need threshold was also not satisfied. We observe different NSR-I for peripheral positions inside and outside the agreeing dyad. For peripheral players who are *included* in the agreeing dyad the NSR-I ranges from 100% with the lowest implemented threshold of 1 point, 99% with 5 points, 94% with 9 points, and to 89% with the highest level of 12 points (see “Tests of difference H3” in S2 File for further analyses). The logistic regression in Table 3 shows the robustness of the effect of the need threshold on need satisfaction.

**Table 3:** Logistic regression of SVO, threshold and other controls on the probability of need satisfaction of the third player.

	Coeff.	S.E.	Lower 0.95	Upper 0.95	Odds Ratio
SVO of network members					
SVO central player	1,2578	0,3135	0,6432	1,8723	3.5175
SVO coalition partner	0,0854	0,212	-0,3301	0,501	1.0892
SVO third player	-0,0876	0,1567	-0,3946	0,2195	0.9161
Period	-0,3521	0,1747	-0,6945	-0,0098	0.7032
Individual threshold of third (ref. = 1)					
Threshold = 5	-0,5901	0,2257	-1,0324	-0,1479	0.5543
Threshold = 9	-1,8599	0,2611	-2,3716	-1,3482	0.2610
Threshold = 12	-2,0505	0,3309	-2,6991	-1,4019	0.1287
Sociodemographic variables of third player					
Female	0,2945	0,19	-0,078	0,667	1.3425
Experimental experience (1 = more than 3)	0,2332	0,2216	-0,2011	0,6674	1.2626
Age (1 = above median of 23 yrs.)	0,2277	0,2065	-0,1771	0,6324	1.2556

The dependent variable of this logistic regression is the probability of the third (i.e. excluded) player having her need threshold satisfied (1) or not (0). We control for the central player’s SVOs and the third player’s own SVO (numeric, all multiplied by 10 for display ease; minimum = -16.26, maximum = 57.83, mean = 20.36), as well as gender (binary; 1 = female), age (binary; 0 = up to 22 years, 1 = 23 years and older) and experimental experience (binary; 0 = 3 or fewer times, 1 = 4 or more times participated in any lab experiment), as these factors can influence bargaining behavior.

N = 546; all scenarios with affluence (i.e. sum of thresholds < available resource), furthermore scenario c0-0-0 is excluded, as there are no thresholds to be satisfied; furthermore, all cases where the individual threshold = 0 are excluded (c5-0-9). Cases without agreement are excluded.

Standard Errors are clustered on the group level of the session, whereby one session consisted of either one or two independent groups of 12 individuals, depending on whether one or both treatments were implemented at the same time.

In contrast, *excluded peripheral players*–third players–have more difficulty getting their need thresholds satisfied. Their NSR-I decreases more rapidly with the threshold level. It drops from 69% at the threshold of 1 point, to 55% at a threshold of 5 points, to 27% at a threshold of 9 points and to 25% at the threshold of 12 points. There is a qualitative and, particularly for the third player, significant difference in the NSR-I for need thresholds surpassing the equal three-way split (i.e., 8 points) compared to those below (supporting *H4*). Below the equal distribution NSR-I decreases with a rising need threshold, but once equality is trespassed, it stagnates at a low level, though not reaching zero.

## Conclusion

Does the normative postulate that needs matter affect human behavior? Testing this proposition is intricate in “natural” environments due to the fundamental ambiguity of the concept of needs. In order to experimentally test the effect of needs in distributive decisions, we operationalized need thresholds in terms of a requirement for access to a next stage in a monetarily incentivized experiment. We explored the effect of heterogeneous need levels in dyadic distributive decisions in a triad on the responsiveness of allocations to threshold levels controlling for social preferences and network positions.

First, we find that need thresholds are effective focal points of individual allocations, in comparison to distributions observed in an environment without such thresholds. Central players in the three-line network do not uniformly use their power to increase their own allocation but behave in line with their social values. In one quarter of all observed decisions, the dyad forgoes half of the available resource in order to lift the third player above the threshold, which means that the power structure is overruled by the norm of need satisfaction in these cases. Second, as expected, the need satisfaction rate declines with increasing thresholds, in line with the expectation that subjects are confronted with a trade-off between their self-interest and their social values. People tilt toward self-interest the more maintaining their social values becomes costly to themselves. Third, this trade-off is substantially more pronounced for the allocation to outsiders. Fourth, the equal distribution marks the point at which the need satisfaction rate of outsiders collapses. Beyond the equal distribution, the principle of need-based justice does not only compete with self-interest, but also conflicts with the principle of equality.

The paper makes three arguments. First, it shows that norms and shared values are relevant in network exchanges and that research designs that restrict the decision space to self-interest by assumption miss an important aspect of human behavior. Second, the results reveal that needs are a highly relevant criterion of distributive decisions in small-scale laboratory societies. And third, it extends our understanding of the conditions under which different principles of justice come into play. In particular, if equality and needs conflict, short-term outcome equality concerns tend to win against individual needs.

We suspect that in this situation, equality is actually used by at least some players as a scapegoat to mask self interest because it is a socially more acceptable reason to deny the satisfaction of needs to those who need more than the equal share. Disentangling these motives, however, must be left to further inquiry using an experimental design that focuses on these differences.

## Materials and methods

### Model

We explore the trade-off between self-interest and justice preferences. We use a general utility function assuming that there exists a payoff distribution embodying a just distribution of payoffs [56]. Without further specifying the properties of this distribution, a vector *U* captures the differences between individual payoff and the payoff perceived as just. Each individual evaluates *U* via a personal weighting function, *f*_*i*_(*U*), which is monotonically increasing in injustice, and a weighting factor, *α*_*i*_, which weights injustice against one’s own payoff *y*_*i*_. The general functional form for evaluating one’s own payoffs with a concern for justice is then: *V*_*i*_(*y*,*U*) = *y*_*i*_−*α*_*i*_*f*_*i*_(*U*).

By avoiding the question of what constitutes justice, this form incorporates justice perceptions such as equality or equity and covers prominent preference functions such as inequality aversion [54, 57]. The satisfaction of needs constitutes an important part of this just distribution. We assume that there exists a threshold vector *T*, which constitutes the need thresholds of each individual in the society, *t*_*i*_ [11, 58, 59]. An individual’s need is satisfied if her payoff is equal to or higher than this threshold, i.e. *s*_*i*_∈{0,1}, where *s*_*i*_ = 1 if *y*_*i*_≥*t*_*i*_ and 0 otherwise. Thus, the need satisfaction vector *S* shows the proportion of individuals in society whose needs are satisfied. Importantly, injustice increases monotonically the fewer needs are satisfied. We assume that any deviation from the just distribution that does not satisfy individual needs affects the weighting function more severely than other deviations. To visualize the effect, we split vector *U* into two parts, one which disregards needs and one which focuses only on needs, *U* ={*U*^¬*S*^∩*U*^*S*^}. For simplicity we assume that the personal weighting function is additive for this relation, i.e. *f*_*i*_(*U*^¬*S*^,*U*^*S*^) = *g*_*i*_(*U*^¬*S*^)+*h*_*i*_(*U*^*S*^). Thus, a utility function which separates the need principle from further injustice takes the following form:

$$V_{i}(y,U^{\neg S},U^{S})=y_{i}-\alpha_{i}g_{i}(U^{\neg S})-\alpha_{i}h_{i}(U^{S})$$ (1)

Satisfying need thresholds by means of the distribution of resources fulfills a minimal justice criterion, which enters the utility function here as a (need) penalty if others’ or own needs are not satisfied. Thus, when individuals allocate payoffs, we expect that (varying) need thresholds pull allocation patterns towards these thresholds, which constitute focal points [60], compared to a situation without needs.

### Experimental design: Additional information

#### Additional measures

Social value orientations, an incentivized, social-psychological construct to measure other-regarding motivations, were measured by means of the SVO slider task [53] prior to the start of the first scenario. In this task, participants are confronted with six different allocation decisions between self and a present but unknown recipient, whereby trade-offs between individual profit maximization, equality, efficiency and altruistic concerns are implemented. One of these six decisions was randomly chosen for payoff, as sender and as recipient. Payoffs from this task were communicated at the end of a session, after completion of the main part of the experiment and the questionnaire. After the seventh round, subjects completed a questionnaire comprising socio-demographic questions. Points were converted to Euros at the end of the experiment.

#### Recruitment method, sample and implementation

The experiment was programmed in zTree [61] and subject recruitment from the university pools was administered by ORSEE [62] and hroot [63]. Experiments were implemented in the VCEE Laboratory at the University of Vienna in 2016 and in the WISO-Experimentallabor at Universität Hamburg in 2017. Both laboratories adhere to the principles of economic experiments and have obtained a waiver from their institutions' ethics commissions (Ethikkommission der Universität Wien, http://ethikkommission.univie.ac.at/; Ethikkommission des Fachbereichs Informatik der Fakultät für Mathematik, Informatik und Naturwissenschaften der Universität Hamburg, https://www.inf.uni-hamburg.de/en/home/ethics.html). They do not admit experiments using deception or that interfere in any other way with the participants' rights. All participants have voluntarily registered for the subject pool and have been informed about the conditions of economic experiments. Students receive invitations to sessions via email. In the present experiment, invitations were restricted to pool members who indicated to understand the local language. 288 individuals participated in total. In both experiments, the resulting sample contained about 60 percent women, average age was about 24 years and participants were in their fifth semester on average. While in Vienna, the average subject had participated in ten experiments before, this number was three in Hamburg (for a more detailed description of the demographic details, see Table A in S1 File). The inclusion of a dummy representing the laboratory in the regression models does not substantively or statistically alter the coefficient estimates (see *Table A in S3 File*). Subjects were randomly seated in computer cubicles upon their arrival and obtained instructions (see S4 File) and answered control questions. Subjects earned on average 22.6 Euros (median = 23.5; range = [8.5; 40]) in about two hours. One scenario was randomly selected and added to the payoff from the SVO task.

#### Details on procedures

If the central player received more than one offer at the same time–one from each player–she could choose between these offers or send counter offers. This constitutes the power of the central position. Players in peripheral network position could only send offers to or receive offers from the central player. They could choose between accepting and not accepting the last offer that they received or send a counter offer to the central player. Accepting an offer was possible by clicking on the offer that was displayed on screen and click the agreement-button. This was also described in the instructions that were handed to the participants. The instructions also included a screen shot of this stage of the experiment. In the practice period subjects had the possibility to familiarize themselves with this mechanism and ask clarifying questions.

## Supporting information

S1 File Supplementary information on sample. Table A. Demographic statistics of subjects in the sample.
(DOCX)

S2 File Additional results. Exemplary effects of differences in thresholds. Tests of difference H3. Tests of difference H4. Further empirical observations. Figure A. Predicted Probabilities (95% conf. int.) of the regression in Table 2. Table A. Logistic regression on NSR-N including control for period. Figure B. Predicted probabilities (95% conf. int.) of regression in Table A in S2 File.
(DOCX)

S3 File Difference in experience between samples. Table A. Logistic regression of NSR-I of third player including lab dummy. Table B. Logistic regression on NSR-N including lab dummy.
(DOCX)

S4 File Experimental instructions (translated from German). (DOCX)

S5 File List of abbreviations and notation. (DOCX)
