# Supplementary material for: The impact of need on distributive decisions: Experimental evidence on anchor effects of exogenous thresholds in the laboratory
Source: PLoS One. 2020 Apr 1;15(4):e0228753. doi: 10.1371/journal.pone.0228753 (PMC7112157; doi:10.1371/journal.pone.0228753)
Supplement: S1 File — Table A. Demographic statistics of subjects in the sample. (DOCX) [file pone.0228753.s001.docx]

# S1 File: Supplementary Information on Sample

## **Table A. Demographic statistics of subjects in the sample**

|  | **VIENNA (n=144)** | **HAMBURG (n=144)** | **Total (n=288)** |
| --- | --- | --- | --- |
| **Sex** |  |  |  |
| Female | 84 (58.3%) | 88 (61.1%) | 172 (59.7%) |
| Male | 60 (41.7%) | 56 (38.9%) | 116 (40.3%) |
| **Age (years)** |  |  |  |
| Mean (SD) | 24.4 (4.47) | 24.0 (7.00) | 24.2 (5.87) |
| Median [Min, Max] | 23.0 [18.0, 45.0] | 22.0 [17.0, 58.0] | 23.0 [17.0, 58.0] |
| **Semester** |  |  |  |
| Mean (SD) | 6.27 (4.05) | 4.81 (4.27) | 5.54 (4.22) |
| Median [Min, Max] | 6.00 [1.00, 22.0] | 3.00 [1.00, 20.0] | 5.00 [1.00, 22.0] |
| **Field of Study** |  |  |  |
| Natural Sciences | 20 (13.9%) | 37 (25.7%) | 57 (19.8%) |
| Technical Studies | 10 (6.9%) | 15 (10.4%) | 25 (8.7%) |
| Economics | 42 (29.2%) | 14 (9.7%) | 56 (19.4%) |
| Social Sciences | 42 (29.2%) | 38 (26.4%) | 80 (27.8%) |
| Human Sciences | 23 (16.0%) | 24 (16.7%) | 47 (16.3%) |
| Life Sciences | 7 (4.9%) | 16 (11.1%) | 23 (8.0%) |
| **Experimental Experience** |  |  |  |
| Mean (SD) | 10.4 (11.9) | 3.31 (4.30) | 6.85 (9.60) |
| Median [Min, Max] | 6.50 [0.00, 100] | 2.00 [0.00, 30.0] | 4.00 [0.00, 100] |
