# Supplementary material for: The impact of need on distributive decisions: Experimental evidence on anchor effects of exogenous thresholds in the laboratory
Source: PLoS One. 2020 Apr 1;15(4):e0228753. doi: 10.1371/journal.pone.0228753 (PMC7112157; doi:10.1371/journal.pone.0228753)
Supplement: S2 File — Exemplary effects of differences in thresholds. Tests of difference H3. Tests of difference H4. Further empirical observations. Figure A. Predicted Probabilities (95% conf. int.) of the regression in Table 2. Table A. Logistic regression on NSR-N including control for period. Figure B. Predicted probabilities (95% conf. int.) of regression in Table A in S2 File. (DOCX) [file pone.0228753.s002.docx]

# S2 File: Additional results

## Exemplary effects of differences in thresholds

*Distribution of profits in scenarios c0-0-0 and c5-12-1:* The mean range of allocated points in these two scenarios is 7.47 and 9.61 points, implying that equality decreases with the introduction of need thresholds. Mean payoff in scenario $c0-0-0$ is 10.92 for the central position and 6.8 averaged over the two peripheral positions. The difference between the powerful position and the peripheral positions is substantial (Wilcoxon rank sum test, z = 5.188, p < 0.001, two-sided), in line with the higher structural power of the central position. Mean payoff in scenario $c5-1-12$ is 10.39 for the central position (threshold of 5). For peripherals, the means are 5.95 (threshold of 1) and 7.66 (threshold of 12), leading to a notable difference of 1.71 points between their allocations (Wilcoxon rank sum test, z = -2.238, p = 0.025, two-sided). The difference in mean payoffs between the central and the peripheral position with a threshold of 1 is substantial and significant (Wilcoxon rank sum test, z = 5.489, p < 0.001, two-sided). This difference to the other peripheral position with a threshold of 12 points is substantially smaller, albeit still significant (Wilcoxon rank sum test, z = 1.981, p = 0.048, two-sided). This difference in points received by either the powerful or the peripheral player with a threshold of 12 points is also notably smaller than it is in the reference scenario $c0-0-0$: 2.73 points compared to 4.38 points. Thus, a higher individual threshold translates into a larger mean allocation to this player.

## Tests of difference H3

The decrease of 1 percentage point (pp) in NSR-I between thresholds 1 and 5 (below equal split) is not substantial (z-test; p = 0.655; 95% CI [-0.001; 0.021]). Somewhat stronger are the decreases in NSR-I of 5pp each between thresholds 5 and 9 (z-test; p = 0.0246; 95% CI [0.003; 0.091]) and between thresholds 9 and 12 (z-test; p = 0.395; 95% CI [-0.060; 0.159]).

## Tests of difference H4

The 14pp decrease between thresholds 1 and 5 is substantial (z-test; p = 0.007, 95% CI [0.040; 0.249]), as is the difference in the 28pp decrease between thresholds 5 and 9 (z-test; p = 0.000; 95% CI [0.170; 0.384]). The difference of 2pp in NSR-I between thresholds 9 and 12, which are both above the equal split, is not substantial and not significant (z-test; p = 0.889, 95% CI [-0.132; 0.181]).

## Further empirical observations

Tables 1 shows the distributions of thresholds – scenarios – in each experiment. Column 3 displays the sum of all thresholds per network. Column 4 refers to the need satisfaction rate at the network level (NSR-N); Column 5 displays the mean range of profits within a network, that is, a measure of inequality of the distribution of the resource. In treatment A, the sum of thresholds is consistently negatively related to the NSR-N. Comparing all scenarios with a sum of 15, $c1-9-5$ deviates to the lower end. It appears that the need level of the central position negatively influences her willingness to attend to others’ needs. The mean range of profits is smallest in the scenario $c5-5-5$, where need thresholds are homogeneous, and largest when the range of thresholds (with a maximum of 12) is largest as well. In treatment B, the NSR-N is negatively related to the sum of thresholds, with one exception, the scenario$c5-5-5$. This deviation may be explained by the fact that in the preceding period the resource was insufficient to satisfy all three need thresholds ($c5-12-12$). Furthermore, it was the final scenario before the end of the session.

## **Figure A. Predicted Probabilities (95% conf. int.) of the Regression in Table 2**


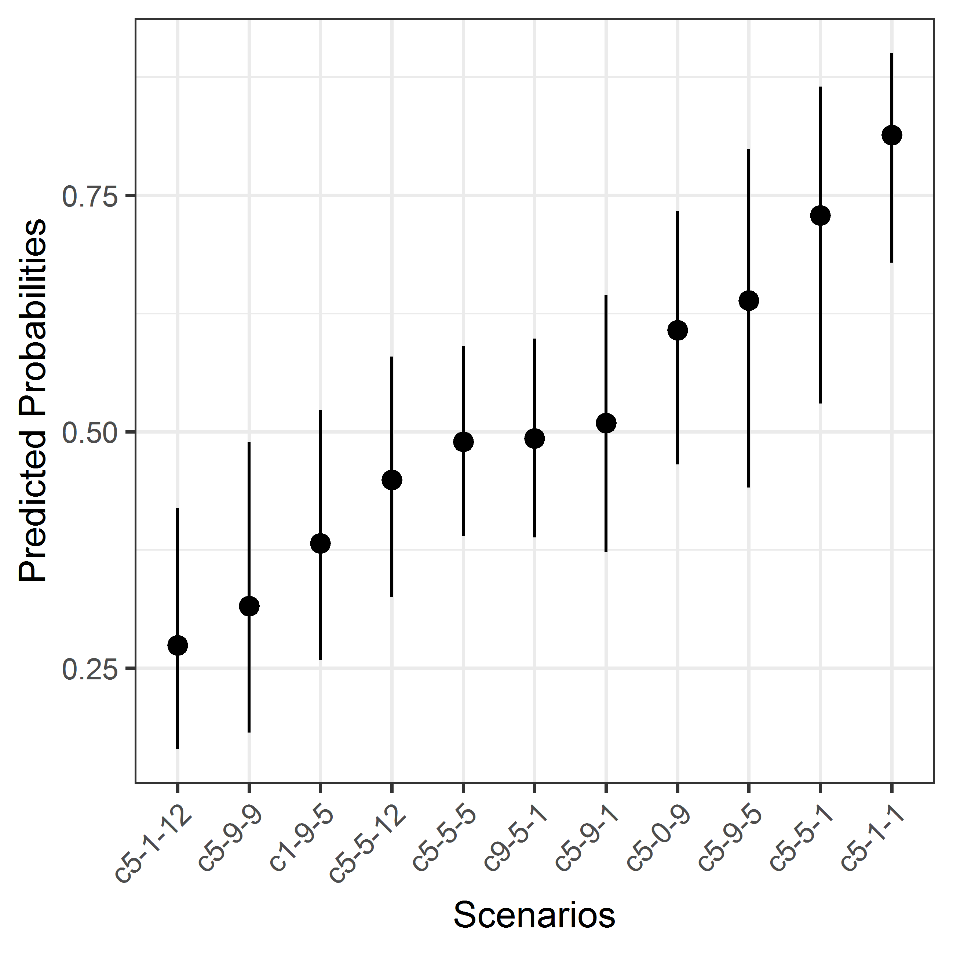


In S1 Figure A predicted probabilities of the treatment effects in the logistic regression in Table 2 are plotted. The sum of SVO of all network members is set at the mean angle of 61.2. Not all scenarios differ substantially from each other, but there are marked differences between groups of scenarios. Note that scenario c5-1-1, in which the sum of thresholds equals 7 points (reference category in regression), has the highest probability of need satisfaction, closely followed by scenario c5-5-1 (sum = 11). In comparison, scenario c5-1-12, which is highly heterogeneous in the distribution of need thresholds and has a sum of 18 points, and scenario c5-9-9, which is less heterogeneous in need threshold distribution but has a sum of 23 points, have the lowest probability of NSR-N reaching 1 (i.e. all three thresholds satisfied). Thus the sum of thresholds and their distribution within the network influence the probability of need satisfaction.

## **Table A. Logistic Regression on NSR-N. Including control for Period**

| *Dependent Variable: NSR-N, 1 = all three thresholds satisfied; 0 = at least one threshold not satisfied* | | | | | |
| --- | --- | --- | --- | --- | --- |
|  | Coefficient | Stand Err | Lower 0.95 | Upper 0.95 | Odds Ratio |
| Sum of SVO in network | 0,5114 | 0,1813 | 0,1561 | 0,8666 | 1,6675 |
| Scenarios (ref. c5-1-1) |  |  |  |  |  |
| c5-5-5 | -1,2936 | 0,4588 | -2,1929 | -0,3944 | 0,2743 |
| c5-0-9 | -0,8918 | 0,522 | -1,915 | 0,1313 | 0,4099 |
| c1-9-5 | -1,8171 | 0,5229 | -2,842 | -0,7922 | 0,1625 |
| c5-1-12 | -2,3089 | 0,5402 | -3,3676 | -1,2502 | 0,0994 |
| c9-5-1 | -1,3898 | 0,4457 | -2,2634 | -0,5162 | 0,2491 |
| c5-9-1 | -1,2414 | 0,4878 | -2,1974 | -0,2854 | 0,289 |
| c5-5-1 | -0,3197 | 0,2386 | -0,7873 | 0,148 | 0,7264 |
| c5-9-5 | -0,8486 | 0,2553 | -1,349 | -0,3482 | 0,428 |
| c5-5-12 | -1,5676 | 0,3605 | -2,2742 | -0,861 | 0,2085 |
| c5-9-9 | -1,9698 | 0,3999 | -2,7535 | -1,1861 | 0,1395 |
| Period | -0,2233 | 0,2455 | -0,7045 | 0,2578 | 0,7998 |

*Note*: N = 576; scenario c5-12-12 is excluded, since NSR-N always < 1; Scenario c0-0-0 is excluded, since NSR-N always = 1. Standard Errors are clustered on the group level of the session, whereby one session consisted of either one or two independent groups of 12 individuals, depending on whether one or both treatments were implemented at the same time.

**Figure B. Predicted Probabilities (95% conf. int.) of the Regression in Table A in S2 File.**


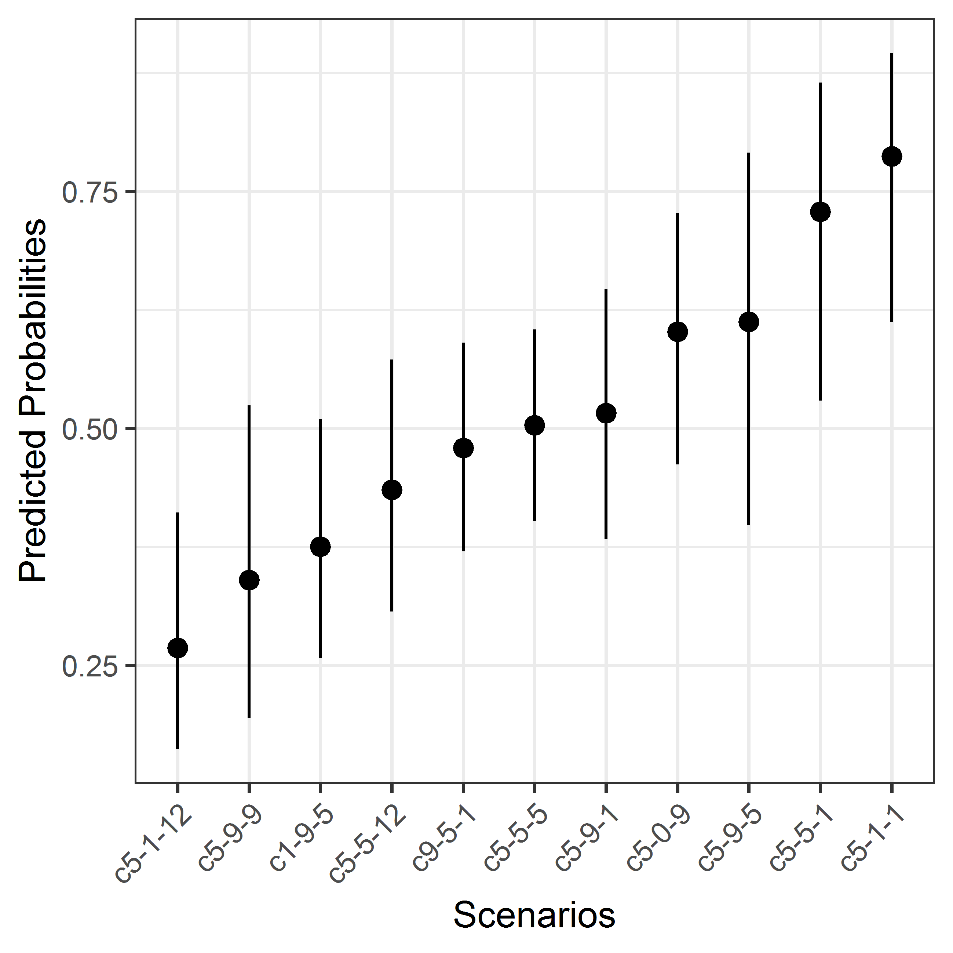


SVO at mean = 61.2, Period at mean = 5. Predicted probabilities are not substantially affected through the inclusion of the time variable.
