# Supplementary material for: The impact of need on distributive decisions: Experimental evidence on anchor effects of exogenous thresholds in the laboratory
Source: PLoS One. 2020 Apr 1;15(4):e0228753. doi: 10.1371/journal.pone.0228753 (PMC7112157; doi:10.1371/journal.pone.0228753)
Supplement: S3 File — Table A. Logistic regression of NSR-I of third player including lab dummy. Table B. Logistic regression on NSR-N including lab dummy. (DOCX) [file pone.0228753.s003.docx]

# S3 File: Difference in experience between samples

## **Table A. Logistic Regression of NSR-I of third including lab-dummy.**

| Dependent Variable: Need of the third player satisfied (no = 0, yes = 1) | | | |  |  |  |
| --- | --- | --- | --- | --- | --- | --- |
|  | Effect | S.E. | Lower 0.95 | Upper 0.95 | Odds Ratio |  |
| SVO of network members |  |  |  |  |  |  |
| SVO central player | 12,256 | 0,3203 | 0,5979 | 18,533 | 34,061 |  |
| SVO coalition partner | 0,0356 | 0,206 | -0,3681 | 0,4394 | 10,363 |  |
| SVO third player | -0,1118 | 0,1728 | -0,4505 | 0,2268 | 0,8942 |  |
| Period | -0,3588 | 0,1736 | -0,6991 | -0,0184 | 0,6985 |  |
| Individual threshold of third (ref. = 1) | |  |  |  |  |  |
| Threshold = 5 | -0,5941 | 0,2246 | -10,342 | -0,154 | 0,552 |  |
| Threshold = 9 | -18,657 | 0,2603 | -23,759 | -13,555 | 0,1548 |  |
| Threshold = 12 | -20,586 | 0,3243 | -26,942 | -1,423 | 0,1276 |  |
| Sociodemographic variables of third player | |  |  |  |  |  |
| Female | 0,314 | 0,1759 | -0,0308 | 0,6587 | 13,688 |  |
| Experimental experience (1= more than 3) | 0,299 | 0,2265 | -0,1448 | 0,7429 | 13,485 |  |
| Age (1 = above median of 23 yrs.) | 0,2321 | 0,2073 | -0,1742 | 0,6384 | 12,612 |  |
| Lab | 0,1888 | 0,4021 | -0,5994 | 0,977 | 12,078 |  |

*Note*: N = 576; scenario c5-12-12 is excluded, since NSR-N always < 1; Scenario c0-0-0 is excluded, since NSR-N always = 1. Standard Errors are clustered on the group level of the session, whereby one session consisted of either one or two independent groups of 12 individuals, depending on whether one or both treatments were implemented at the same time.

## **Table B. Logistic regression on NSR-N including lab-dummy**

| Dependent Variable: NSR-N = 1 (all three thresholds sastisfied); 0 (not all thresholds satisfied) | | | | | |
| --- | --- | --- | --- | --- | --- |
|  | Effect | S.E. | Lower 0.95 | Upper 0.95 | Odds Ratio |
| Sum of SVO in network | 0,4375 | 0,1811 | 0,0825 | 0,7924 | 15,488 |
| Scenarios (ref. c5-1-1) |  |  |  |  |  |
| c5-5-5 | -16,815 | 0,3654 | -23,977 | -0,9654 | 0,1861 |
| c5-0-9 | -1,285 | 0,48 | -22,258 | -0,3441 | 0,2767 |
| c1-9-5 | -22,088 | 0,4403 | -30,718 | -13,459 | 0,1098 |
| c5-1-12 | -27,053 | 0,4712 | -36,288 | -17,818 | 0,0669 |
| c9-5-1 | -17,524 | 0,4017 | -25,397 | -0,9651 | 0,1734 |
| c5-9-1 | -16,855 | 0,4248 | -25,181 | -0,8529 | 0,1854 |
| c5-5-1 | -0,4865 | 0,1813 | -0,8417 | -0,1312 | 0,6148 |
| c5-9-5 | -0,9016 | 0,25 | -13,915 | -0,4117 | 0,4059 |
| c5-5-12 | -16,721 | 0,337 | -23,326 | -10,117 | 0,1878 |
| c5-9-9 | -22,389 | 0,2231 | -26,763 | -18,016 | 0,1066 |
| Lab | 0,3923 | 0,268 | -0,133 | 0,9176 | 14,804 |

*Note:* The dependent variable of this logistical regression is the probability of the third (i.e. excluded) player having her need threshold satisfied (1) or not (0). We control for the central player’s SVOs and the third player’s own SVO (numeric, all multiplied by 10 for display ease; minimum = -16.26, maximum = 57.83, mean = 20.36), as well as gender (binary; 1 = female), age (binary; 0 = up to 22 years, 1 = 23 years and older) and experimental experience (binary; 0 = 3 or fewer times, 1 = 4 or more times participated in any lab experiment), as these factors can influence bargaining behavior. N = 546; all scenarios with affluence (i.e. sum of thresholds < available resource), furthermore scenario c0-0-0 is excluded, as there are no thresholds to be satisfied; furthermore, all cases where the individual threshold = 0 are excluded (c5-0-9). Cases without agreement are excluded. Standard Errors are clustered on the group level of the session, whereby one session consisted of either one or two independent groups of 12 individuals, depending on whether one or both treatments were implemented at the same time.
