# Supplementary material for: The impact of need on distributive decisions: Experimental evidence on anchor effects of exogenous thresholds in the laboratory
Source: PLoS One. 2020 Apr 1;15(4):e0228753. doi: 10.1371/journal.pone.0228753 (PMC7112157; doi:10.1371/journal.pone.0228753)
Supplement: S4 File — (DOCX) [file pone.0228753.s004.docx]

# S4 File: Experimental instructions (translated from German)

**Welcome to the experiment**

**and thank you for your participation!**

Please do not talk to other participants during the experiment!

In this experiment you and all other participants have to decide on several occasions. Your payoff depends on the decisions of the other participants as well as your own decisions. The decisions are the same for all participants.

Please read the instructions carefully and take your time on your decisions.

**Please do not ask your questions openly! Please raise your hand if you have any questions. One of the laboratory personnel will come to you to answer your questions individually. Also, you are not allowed to communicate with other participants during the experiment. It is important for the scientific value of the experiment to abide by this rules.**

You will receive your payoff after the experiment anonymously and in private. You and the other participants will not be informed about the interaction partners or anyone’s payoff, during or after the experiment.

Throughout the experiment we speak of **tokens** instead of real money. After the experiment the tokens you earned will be converted to real money at the rate of:

**1 tokens = 1 Euro**

**1 token = 100 token-cents**

Each participant has the potential to earn 22 tokens (22 Euros) on average, depending on own and other’s decisions. The experiment will last about 1.5 hours on average.

You cannot influence the duration of the experiment with a quick decision, as you have to wait for the other participants to finish.

**Experiment 1**

In to following you will be confronted with several decision situations.

**First Situation:**

In this experiment you have to decide on the allocation of points between yourself and another participant on several occasions. You are offered to choose the allocations you prefer most from 9 options on 6 different occasions. At the end of the experiment one of this 6 decisions is chosen randomly and paid to you and a randomly matched partner. You will also receive the payoff of the allocation some other participant chose.

**Profit (First situation) = own decision + decision of one other participant**

**Second Situation:**

In this experiment you have to decide between two payoff variants under different circumstances in 10 situations. One of you choices will be chosen randomly for your payoff.

**Profit (Second situation) = own decision (randomly chosen lottery)**

**Profit (Experiment 1) = Profit (First situation) + Profit (Second situation)**

You will be informed about the payoff of experiment 1 at the end of the whole experimental session.

**Experiment 2**

For this experiment you will be matched into groups of three people. In total this experiment has **7 periods.** At the beginning you are allocated to one position in the group. You will remain in this position for the entire experiment (labelled with colors red, green, and blue). In each period you are matched with a new group (meaning two new group members). You are also randomly assigned **threshold**. This happens to all participants.

**Each period has two parts (part A and B)**. In the first part you can communicate with the members of your group and come to an agreement about the allocation of 24 tokens. If you manage to obtain enough points to overcome the threshold that was assigned to you, you may earn additional profit in the third part of the experiment.

**Part A: Allocation of points**

In the second part of the experiment you can communicate with your group members about the allocation of 24 tokens. Depending on how you are connected to the members of the group you will see either one or two windows on your computer screen in which you can communicate with one other group member individually. In each period you will receive information about the connections within your group. The thresholds that were assigned to you and the others are displayed as numbers next to the colored circles that mark the positions in the group.

You can communicate using the windows on your screen, in which you can **send offers about the allocation of 24 tokens** back and forth. You can send and receive offers from the participants you are connected with. After **one minute** you can accept the last offer that was sent. Only one offer can be accepted per period. You have **three minutes** to find an agreement. If you do not agree, all group members receive 0 points. Only one agreement is possible. If your two fellow group members manage to agree, you will be informed about their agreement subsequently. The first agreement made between two participants is also valid for the whole group.

**If you manage to obtain enough tokens to satisfy the threshold that was assigned to you in a period you can earn additional profit in the third part of this experiment.** The thresholds are randomly allocated each period and thus can change each period. The thresholds are common knowledge within a group.

**Part B: Tasks**

If you managed to obtain enough points to satisfy your threshold **you can earn additional money in this part of the experiment by completing different real effort tasks**. The points you add in this part are added to the points you obtained in part 2. There are different tasks to be completed, such as addition or multiplication of numbers, answering quiz questions. The tasks can be of varying difficulty and the profit to be earned is related to the level of difficulty. At the end of each period you will be informed about the tokens you earned in this period.

If you did not earn enough points to satisfy you threshold, you cannot earn additional profit, but you can still participate in the completion of the tasks. In this case you receive the tokens you obtained in part 2.

Example 1: You threshold to participate in part 3 is 5 tokens. Your group agreed that you should receive 8 tokens. Thus you can participate in part three and earn additional tokens. In this period you earn 8 tokens plus anything you earned in part 3.

Example 2: You threshold to participate in part 3 is 5 tokens. Your group agreed that you should receive 4 tokens. Thus you cannot earn additional profit in part 3. Your profit of this period equals 4 tokens.

**Payoff of experiment 2**

For payoff only one of the 7 periods is relevant. One period will be randomly drawn for payoff at the end of the experiment. You will receive the payoff of this period and your points will be converted to Euros.

At the beginning of Experiment 2 we ask you to complete some comprehensive questions and to state your preference about some exemplary distributions. Subsequently all participants will complete a trial period in order to familiarize yourself with the procedure. This period is not relevant for payoff.

**Conclusion**

The tokens you earned in experiment 1 and experiment 2 will be added and converted. At the end of the experiment you will receive the payoff you earned in both experiments.

At the end of the experiment you will find a short questionnaire. Please fill in the questionnaire. You will receive 4 tokens for the completion of the questionnaire.

On the next page you will find a summary of experiment 2.


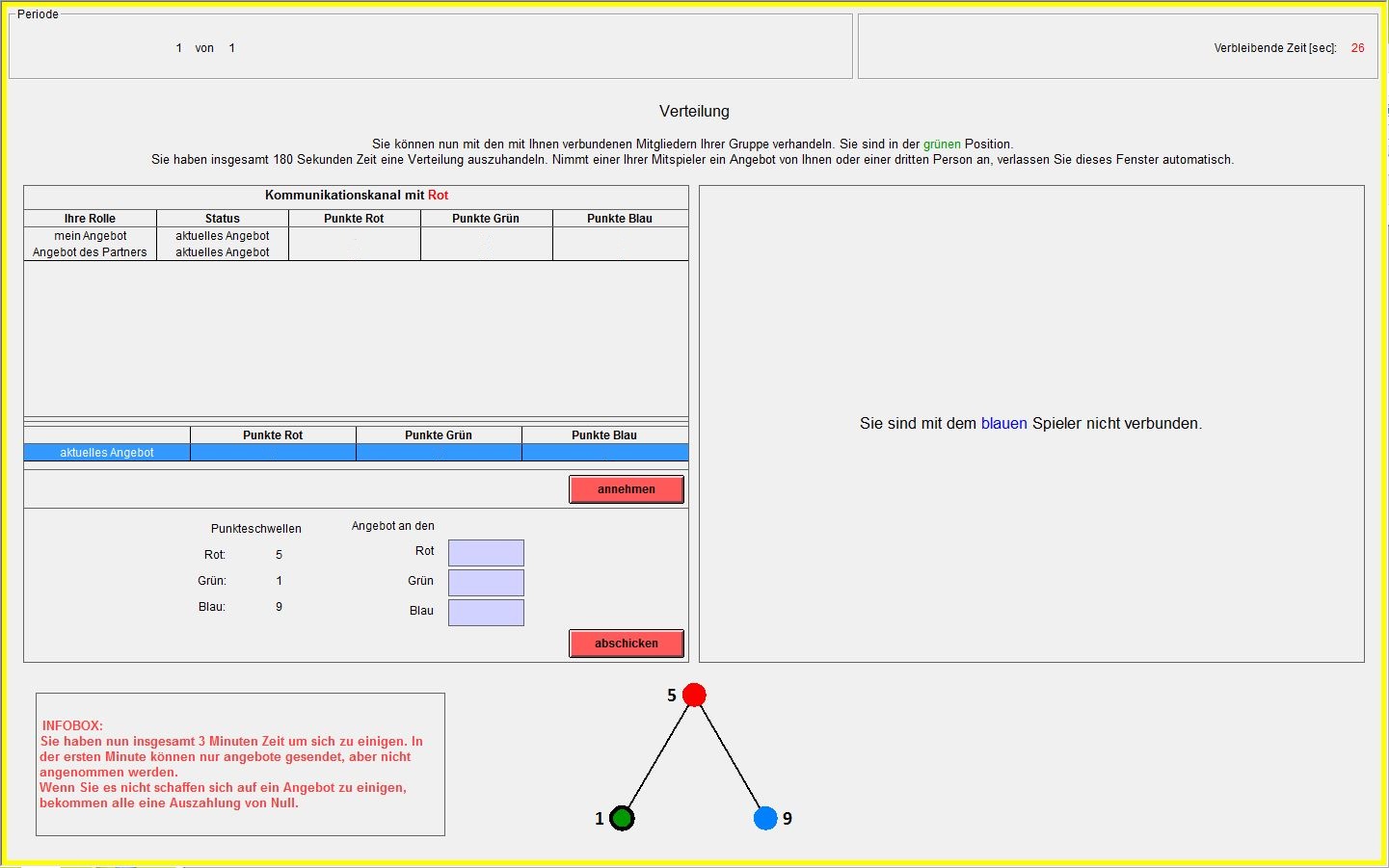


**INFOBOX:**

You have three minutes to find an agreement. In the first minute you can only send, but not accept offers. If no agreement is found, all group members receive 0 points.

Here you can see your position and the ways of communication within your network. Your position: green.

The black number indicates this participants‘ threshold.

Angebot

?

?

?

?

?

?

?

?

?

?

?

?

Here you can see your own offers and the offers of your partner

You have to highlight the offer you want to accept with a click.

Here you can send offers to the red player.
