# Supplementary material for: The impact of need on distributive decisions: Experimental evidence on anchor effects of exogenous thresholds in the laboratory
Source: PLoS One. 2020 Apr 1;15(4):e0228753. doi: 10.1371/journal.pone.0228753 (PMC7112157; doi:10.1371/journal.pone.0228753)
Supplement: S5 File — (DOCX) [file pone.0228753.s005.docx]

# S5 File: List of abbreviations and notation

## Abbreviations

| A-C-B | Positions in the implemented network, whereby C as central player is in the strong power position and A and B as peripheral players are in a weak power position. |
| --- | --- |
| St1, St2, St3 | Stage one, two or three of the conducted experiment. |
| NSR | Need satisfaction rate. |
| NSR-I | Need satisfaction rate on an individual level, i.e. the proportion of individuals whose needs are satisfied for a specific individual threshold. |
| NSR-N | Need satisfaction rate on a network level, i.e. the proportion of need thresholds that are satisfied in the network. |
| SVO | Social value orientation. |
| N | Number of observations. |

## Notation

| *n* | Number of individuals in the network or the group. |
| --- | --- |
| *U* | Vector that captures the difference between individual payoffs and just payoffs for each member *1* to *n*. |
| *f_i_(U)* | Function of individual *i* that weights the injustice within the utility function. |
| *y_i_* | Payoff of individual *i*. |
| *α_i_* | Factor of individual *i* that weights the own payoff against perceived injustice in the utility function. |
| *V_i_(∙)* | Utility function of individual *i*. |
| *t_i_* | Need threshold of individual *i*. |
| *T* | Vector consisting of all need thresholds *t_i_* of members 1 to *n*. |
| *s_i_* | Individual need satisfaction, which is equal to 1 if *y_i_* ≥ *t_i_* and 0 otherwise. |
| *S* | Vector displaying whether the individual thresholds of members 1 to *n* are satisfied. |
| *U^⌐S^* | Vector that captures the difference between the actual payoff distribution and the just distribution without considering individual needs. |
| *U^S^* | Vector that captures the proportion of needs that are *not* satisfied, which is equal to 1-S. |
| *g_i_(U^⌐S^)* | Function of individual *i* that weights the perceived non-need related injustice, such as inequality concerns. |
| *h_i_(U^S^)* | Function of individual *i* that weights the need related injustice, i.e. weights the need thresholds that are not satisfied. |
